# Re-evaluating trainee experience of involvement in serious incidents – has anything changed?

**DOI:** 10.1192/bjo.2021.837

**Published:** 2021-06-18

**Authors:** Glori-Louise de Bernier, Alice Debelle, Lauren Waterman, Marilia Calcia

**Affiliations:** South London and Maudsley NHS Foundation Trust

## Abstract

**Aims:**

To complete an audit cycle to investigate: trainees’ experiences of SI involvement since 2017, perceptions of current support systems and trust facilitation of learning from SIs and the impact of the interventions implemented following the 2017 survey.

**Background:**

In 2017, data were collected from trainees working in psychiatry within two London trusts to examine the nature of their involvement in serious incidents (SIs), their experience of the process following an SI and their knowledge of the support systems available to them. Due to concerning results from this, several interventions were put in place in accordance with trainees’ suggestions.

**Method:**

Cross-sectional surveys were e-mailed to trainees of all grades in July 2019, including GP and foundation doctors, working within two mental health trusts. These built upon the 2017 surveys, additionally enquiring about demographic information and the personal and training consequences of SIs on trainees.

**Result:**

61 (15% of all trainees) returned the survey with 41 (67%) respondents unable to recall any SI related teaching during induction and 47 (77%) not having received a written guidance document on SI procedures.

24 (39%) had been involved in an SI. Only half felt adequately supported by the trust at internal investigation. Knowledge of the available internal and external sources of support ranged from 38-71% however these sources were rarely utilised. 12 (60%) trainees did not feel that learning had been facilitated following an SI and almost none had been informed of internal investigation outcomes.

Respondents who gave a low (1-4/10) rating of support from their NHS Trust were more likely to have been informed about the incident in person, been invited to team-based support or been aware of the variety of sources of support available, when compared with respondents who scored their Trust support more highly. Suggestions for improvements made by trainees included opportunities to observe coroners’ inquests and a peer support scheme from colleagues with experience of SI involvement.

**Conclusion:**

Unfortunately, trainees did not report much improvement in their experiences compared those in the 2017 survey, and a large proportion continued to feel unsupported. Interventions had not been as widely circulated as intended and only half of trainees had been invited to team-based support. Possible further interventions include increasing email communication to trainees following SIs and setting up a peer support scheme. We are in the process of organising a coroner's inquest observation programme for trainees.

